# Comparison of the Mental Disorders Among Families of ISIS Captives Residing in Sulaymaniyah With the Native Population in 2023: A Cross-sectional Study

**DOI:** 10.34172/aim.2024.21

**Published:** 2024-03-01

**Authors:** Bakhtyar Zrar Rahman, Mansour Bayrami, Khalil Esmaielpour

**Affiliations:** ^1^Faculty of Educational Sciences and Psychology, University of Tabriz, Tabriz, Iran; ^2^Department of Psychology, Faculty of Educational Sciences and Psychology, University of Tabriz, Tabriz, Iran; ^3^Department of Psychology, Faculty of Psychological Education, University of Tabriz, Tabriz, Iran

**Keywords:** 90-R-SCL, Families of ISIS war captives, Mental disorders, Residents of Sulaymaniyah

## Abstract

**Background:**

This study aimed to compare the level of mental disorders among families of ISIS captives residing in Sulaymaniyah with the native population in 2023.

**Methods:**

In the present descriptive-analytical cross-sectional study, a total of 383 ISIS captives by census aged 18-60 years were selected, along with an equal number of matched native individuals from Sulaymaniyah in terms of demographic characteristics. The data collection tool was the SCL-90 questionnaire. The 90-R-SCL interview and test were used to assess the level of symptoms related to mental disorders.

**Results:**

The results showed statistically significant differences between ISIS captives and the native population in terms of the total psychological disorder mean score (2.54±0.30 vs. 1.52±0.16; *P*<0.001), Global Severity Index (GSI) (253.40±32.82 vs. 137.03±14.74; *P*<0.001) and the mean scores of the dimensions of psychological disorder including: physical complaint (2.52±0.45 vs. 1.67±0.54; *P*<0.001), obsessive compulsive disorder (2.51±0.43 vs. 1.50±0.44; *P*<0.001), disorder in interpersonal relationships (2.55±0.44 vs. 19.10±0.29; *P*<0.001), depressive disorder (2.60±0.41 vs. 1.60±0.55; *P*<0.001), anxiety disorder (2.50±0.41 vs. 12.10±0.29; *P*<0.001), aggression disorder (2.55±0.49 vs. 19.10±0.40; *P*<0.001), morbid fear disorder (2.55±0.45 vs. 1.48±0.45; *P*<0.001), paranoid ideation disorder (2.49±0.55 vs. 1.39±0.40; *P*<0.001), and psychotic disorder (2.47±0.43 vs. 1.52±0.57; *P*<0.001).

**Conclusion:**

The findings of this study suggest that ISIS captives suffer from multiple psychological disorders, and the presence of more severe mental disorders among this population necessitates comprehensive psychiatric and psychological services for them.

## Introduction

 The structure of modern societies has placed families at risk of various mental disorders, making it nearly impossible for them to be immune to psychological pressures.^1^ Nowadays, with the existing societal problems, families are directly and indirectly exposed to multiple psychological pressures that can lead to mental disorders. This issue is particularly prominent for families experiencing unique circumstances such as imprisonment, captivity, and war.^2^ Between 2014 and 2017, the International Organization for Migration estimated that with the attack of ISIS terrorists and the fall of cities in northern and western Iraq, 5.8 million residents of these areas were relocated to refugee camps. According to official statistics from the Iraqi Ministry of Migration and Displacement, there are currently 465 000 people in refugee camps across Iraq.^2,3^

 The stages of captivity described by the authors consist of three stages: pre-captivity, the duration of captivity, and post-captivity. The first stage, called “apprehension,” begins from the moment the prisoner encounters the enemy.^4^ In this stage, the individual experiences a lack of knowledge about how the enemy will treat them, solitary living, physical torture, and intense environmental pressures such as deprivation of food, shelter, and clothing.^5^ The second stage marks the uniformity of captivity. Factors contributing to physical and psychological pressure in this stage include continuous deprivation of food, arbitrary torture and beatings, the discomfort of individuals in the prison environment, disruption of previous lifestyle routines, and long-term ambiguity and uncertainty.^6^ If a person fails to adopt a clear life pattern in captivity and adapt to the environment, any of these stages will be sufficient to drive them towards psychological collapse.^7^ The third stage is the freedom of the prisoner after years of pressure and hardship. The individual’s perception of their own freedom, social support, and exposure to distressing news such as the death of friends, relatives, children, or spouses, and changes in social conditions are major factors that can lead to the development of psychological disorders, social maladjustment, and depression upon release. Social maladjustment may be accompanied by anxiety and can manifest in various degrees.^8,9^ Depression is a disorder characterized by a major change in mood and encompasses feelings of mild sadness to severe despair. Moreover, there are noticeable changes in behavior, perception, thinking, performance, and physiological functions such as fatigue, loss, and incapacity for decision-making, disregard for appearance and clothing, physical symptoms such as loss of appetite, constipation, sleep disturbances, problem-solving difficulties, loss of meaning in life, and boredom are signs of depression.^10^ During the ISIS crisis from 2014 to 2017, a new wave of social and psychological conflicts occurred in the region. This crisis forced thousands of internally displaced persons from other parts of Iraq to flee their homes and seek security and refuge in the Kurdistan region.^11,12^ The Garmian region in eastern and southeastern Kurdistan, due to its shared borders with Arabic-speaking cities in Iraq, has been one of the destinations for a large number of internal migrants. As a result, more than 1,756 families live in four refugee camps in this region, and many others have settled informally in urban areas and villages.^13^

 Numerous studies have been conducted on the impact of captivity on the mental and physical health of prisoners. Some of these studies have shown that individuals who have been in prisons, refugee camps, or other captive situations are usually exposed to severe stressors that can have serious consequences for their mental and physical health. In line with this, and providing a clear picture of the psychological status of families of ISIS captives in Iraq, this study aimed to compare with the native population, the level of psychological disorders among families of ISIS captives residing in Sulaymaniyah.

## Materials and Methods

 The current study utilized a cross-sectional descriptive-analytical design.

###  Implementation

 After necessary coordination with the officials of the refugee camps, a total of 383 individuals selected by census method from one camp participated in the study. The criterion for inclusion in the research was the cooperation of the refugees. The research utilized a questionnaire on personal information and the 90-SCL test. The reliability of this 90-item test, which includes 9 categories of psychiatric symptoms such as somatic complaints, obsession-compulsion, interpersonal sensitivity, depression, anxiety, aggression, phobic anxiety, paranoid ideation, and psychoticism, has been previously confirmed. The questionnaires were completed by a psychologist in the presence of a psychiatrist and a translator, if necessary, and in a face-to-face manner.

###  The Data Collection Tools

 The data collection tool in this study was a questionnaire. The questionnaires were completed by a psychologist in the presence of a psychiatrist and a translator, if necessary, in a face-to-face manner. Patient information was coded to maintain confidentiality. In this study, the questionnaire on demographic information and the Symptom Checklist-90 (SCL-90) test were used. This short personality test not only diagnoses psychiatric patients but is also used successfully for screening alcohol and substance addicts, sexual dysfunctions, cancer patients, patients with severe physical ailments, individuals in need of counseling, or for screening purposes. The initial form of this questionnaire (SCL-90) was designed by Derogatis, Lipman, and Covi (1973) to assess the psychological aspects of physical and mental patients. In 1984, Derogatis and colleagues revised the questionnaire and published its final version, called the Symptom Checklist-90-Revised (SCL-90-R). This short-answer questionnaire consists of 90 items with five options (not at all = 0; a little = 1, moderately = 2; much = 3, very much = 4). The content of this test measures nine different dimensions, including: physical complaint, obsessive compulsive disorder, disorder in interpersonal relationships, depressive disorder, anxiety disorder, aggression disorder, morbid fear disorder, paranoid ideation disorder, and psychotic disorder. We also translated and used this questionnaire in Persian and Kurdish languages with the help of an expert who was proficient in translation work.

###  Data Analysis

 The analysis included a descriptive and an analytical part. In the data description section, quantitative variables were reported with the average score and standard deviation, as well as the lowest and highest score. In the analysis section, depending on the nature of the compared variables and establishing the defaults, the appropriate test was used. To measure the normality of the data, the Kolmogorov-Smirnov test was used, and the statistical test was also used based on the type of test. In order to compare the average scores between two groups, due to the rank nature of the data and the lack of presumptions of parametric tests, the non-parametric equivalent of the *t* test, i.e. the Mann-Whitney U-test, was used. A significance level of less than 5% was considered and all analyses were performed in Stata v14.

###  Ethical Considerations

 The researcher maintained the identifying information of the study’s participants. All participants provided written informed consent prior to the distribution of the questionnaires.

## Results

 The psychological status of ISIS captives, based on the results of the SCL-90 questionnaire, has been presented in Table 1.

**Table 1:** Psychological Status Assessment of Families of ISIS Captives in the Present Study

**Variables**	**Number**	**Mean**	**Standard Deviation**	**Minimum**	**Maximum**
Physical complaint	383	2.52	0.45	0.92	3.67
Obsessive compulsive disorder	383	2.51	0.43	1.20	3.70
Disorder in interpersonal relationships	383	2.56	0.44	1.11	3.56
Depressive disorder	383	2.60	0.41	0.85	3.69
Anxiety disorder	383	2.50	0.41	1.30	3.50
Aggression disorder	383	2.55	0.49	0.83	3.67
Morbid fear disorder	383	2.55	0.45	1.43	3.86
Paranoid ideation disorder	383	2.50	0.55	0.50	3.83
Psychotic disorder	383	2.48	0.43	1.20	3.70
total score	383	2.54	0.30	1.61	3.37

 The results of Table 1 show that all dimensions of the questionnaire, except for psychosis, scored above 2.5, indicating significant psychiatric disorder symptoms in these dimensions. It should be noted that psychosis encompasses a range of symptoms, from mild alienation to full-blown symptoms of schizophrenia such as delusions and thought disorganization. It includes a gradual progression of disturbance, from mild estrangement to acute psychosis, and had an average score very close to 2.5, specifically 2.48 ± 0.43. The highest score among ISIS captives was related to depression (2.60 ± 0.41), which included symptoms such as depressed mood, loss of interest in life’s pleasures, lack of motivation and vital energy, feelings of hopelessness, suicidal thoughts, and other cognitive and physical aspects. Additionally, the overall mean score of the questionnaire was 2.54 ± 0.30, indicating the presence of significant psychiatric disorder among ISIS captives. Among ISIS captives, the lowest score obtained was related to paranoid ideation, while the highest score obtained was related to fear and aggression disorders.

 The psychological status of native individuals, based on the results of the SCL-90 questionnaire, is presented in Table 2.

**Table 2:** Psychological Status Assessment of Native Individuals in the Present Study

**Variables**	**Number**	**Mean**	**Standard Deviation**	**Minimum**	**Maximum**
Physical complaint	383	1.67	0.54	1	3
Obsessive compulsive disorder	383	1.50	0.44	1	3
Disorder in interpersonal relationships	383	1.19	0.29	1	3
Depressive disorder	383	1.60	0.55	1	3
Anxiety disorder	383	1.12	0.29	1	3
Aggression disorder	383	1.19	0.40	1	3
Morbid fear disorder	383	1.48	0.45	1	2.10
Paranoid ideation disorder	383	1.39	0.40	1	3
Psychotic disorder	383	1.52	0.57	1	3
Total score	383	1.52	0.16	1.14	2.05

 The results of Table 2 show that individuals from the native population scored below 2.5 on average in all dimensions, indicating the absence of significant psychiatric disorder symptoms in all dimensions. Physical complaints, which are indicative of distress related to the perception of unhealthy bodily functioning and manifest as complaints about cardiovascular, gastrointestinal, and respiratory systems, had the highest average of 1.67 ± 0.54 among native individuals. The lowest average, 1.19 ± 0.29, among native individuals was related to interpersonal sensitivity, which emphasizes individual sensitivity in interpersonal relationships, feelings of inadequacy and inferiority, particularly in comparison to others. It includes self-deprecation, feelings of restlessness, perceived lack of understanding by others, feelings of embarrassment and discomfort in front of the opposite sex, and a sense of not being treated as a friend by others, contributing to interpersonal sensitivity. Additionally, the overall mean score of the questionnaire was 1.52 ± 0.16, indicating the absence of significant psychiatric disorder among native individuals.

 The comparison of the various dimensions of the mental disorders among families of ISIS captives residing in Sulaymaniyah with the native population has been shown in Table 3.

**Table 3:** Comparison of Various Dimensions of Mental Disorders Among Families of ISIS Captives Residing in Sulaymaniyah With the Native Population in the Present Study

**Dimensions of the Mental Disorders**	**Groups**	**Mean**	**Standard Deviation**	***P*** ** Value**
Physical complain	Native people	1.67	0.54	< 0.001
	ISIS prisoners	2.52	0.45	
Obsessive compulsive disorder	Native people	1.50	0.44	< 0.001
	ISIS prisoners	2.51	0.43	
Disorder in interpersonal relationships	Native people	1.19	0.29	< 0.001
	ISIS prisoners	2.55	0.44	
Depressive disorder	Native people	1.60	0.55	< 0.001
	ISIS prisoners	2.60	0.41	
Anxiety disorder	Native people	1.12	0.29	< 0.001
	ISIS prisoners	2.50	0.41	
Aggression disorder	Native people	1.19	0.40	< 0.001
	ISIS prisoners	2.55	0.49	
Morbid fear disorder	Native people	1.48	0.45	< 0.001
	ISIS prisoners	2.55	0.45	
Paranoid ideation disorder	Native people	1.39	0.40	< 0.001
	ISIS prisoners	2.49	0.55	
Psychotic disorder	Native people	1.52	0.57	< 0.001
	ISIS prisoners	2.47	0.43	

 The results of Table 3 show that the average score of somatic complaints among native individuals was 1.67 ± 0.54, while it was 2.52 ± 0.45 among ISIS captives. Based on the results, there was a statistically significant difference in the average scores of somatic complaints between native individuals and ISIS captives, and ISIS captives obtained a higher average score (*P* < 0.001). The average score of obsessive-compulsive disorders among native individuals was 1.50 ± 0.44, while it was 2.51 ± 0.43 among ISIS captives. Based on the results, there was a statistically significant difference in the average scores of OCD between native individuals and ISIS captives, and ISIS captives obtained a higher average score (*P* < 0.001). The average score of interpersonal sensitivity among the native population was 1.19 ± 0.29, while it was 2.55 ± 0.44 among ISIS captives. According to the results, there was a statistically significant difference in interpersonal sensitivity between the native population and ISIS captives, and ISIS captives obtained a higher average score (*P* < 0.001). The average score of depression among the native population was 1.60 ± 0.55, while it was 2.60 ± 0.41 among ISIS captives. According to the results, there was a statistically significant difference in depression between the native population and ISIS captives, and ISIS captives obtained a higher average score (*P* < 0.001). The average score of anxiety among the native population was 1.12 ± 0.29, while it was 2.0 ± 50.41 among ISIS captives. According to the results, there was a statistically significant difference in anxiety between the native population and ISIS captives, and ISIS captives obtained a higher average score (*P* < 0.001). The average score of aggression among the native population was 1.19 ± 0.4, while it was 2.55 ± 0.49 among ISIS captives. According to the results, there was a statistically significant difference in aggression between the native population and ISIS captives, and ISIS captives obtained a higher average score (*P* < 0.001). The average score of pathological fear among the native population was 1.48 ± 0.45, while it was 2.55 ± 0.45 among ISIS captives. According to the results, there was a statistically significant difference in pathological fear between the native population and ISIS captives, and ISIS captives obtained a higher average score (*P* < 0.001). The average score of paranoid ideations among the native population was 1.39 ± 0.40, while it was 2.49 ± 0.55 among ISIS captives. According to the results, there was a statistically significant difference in paranoid ideation between the native population and ISIS captives, and ISIS captives obtained a higher average score (*P* < 0.001). The average score of psychological distress among the native population was 1.52 ± 0.57, while it was 2.47 ± 0.43 among ISIS captives. According to the results, there was a statistically significant difference in psychological distress between the native population and ISIS captives, and ISIS captives obtained a higher average score (*P* < 0.001).

 The results of the comparison of the overall score of psychological disorders between families of ISIS captives and the native population have been presented in Table 4.

**Table 4:** Comparison of Overall Psychological Disorder Scores Between ISIS Captives and Native Individuals

**Dimensions of the Mental Disorders**	**Groups**	**Mean**	**Standard Deviation**	***P*** ** Value**
Overall psychological disorder score	Native people	1.52	0.16	< 0.001
	ISIS prisoners	2.54	0.30	

 The results of Table 4 show that the average score of the overall psychological disorder in the native population was 1.52 ± 0.16, while it was 2.54 ± 0.30 among ISIS captives. According to the results, there was a statistically significant difference in the overall score of psychological disorders between the native population and ISIS captives, and ISIS captives obtained a higher average score (*P* < 0.001).

 The results of the comparison of the Global Severity Index (GSI) score of symptoms between the native population and ISIS captives have been presented in Table 5.

**Table 5:** Comparison of Overall Psychological Disorder Scores Between ISIS Captives and Native Individuals

**Dimensions of the Mental Disorders**	**Groups**	**Mean**	**Standard Deviation**	***P*** ** Value**
GSI	Native people	137.03	14.74	< 0.001
	ISIS prisoners	253.34	32.82	

GSI, Global Severity Index.

 The results of Table 5 showed that the average score of psychopathological symptoms in native individuals was 14.74 ± 137.03, while it was 32.82 ± 253.34 among ISIS captives. According to the results, a statistically significant difference was observed between native individuals and ISIS captives in terms of the average Global Severity Index (GSI), with ISIS captives having higher scores.

## Discussion

 Mental disorders are believed to be the primary cause of the rise in problems and impairments brought on by poor health around the world and have raised numerous concerns for health systems, specialists, and policy makers in the sector.^14^ People’s mental health is linked to their emotional, psychological, social, and even physical well-being, according to several published studies.^15-17^ In the current study, all mean scores for assessing the psychological status of families of ISIS captives were greater than those of the native people (Table 1 vs. Table 2). This research highlights the need for monitoring people’s psychological state after experiencing an unexpected and tragic event. According to numerous published studies, people who suffer unanticipated catastrophic events will have psychological alterations both immediately and later on, which will have an impact on their health.^18,19^

 Our results (Table 3) showed that various dimensions of the mental disorders such as physical complain, interpersonal relationships, obsessive compulsive disorder, depressive, anxiety, aggression, paranoid ideation, psychotic and fear disorders among families of ISIS captives residing in Sulaymaniyah were statistically significantly different compared to the native population. Consistent with these findings, the results of several studies such as those by Hao et al,^20^ Bakioğlu et al,^21^ Główczyński et al^22^ and Bellapigna & Kalibatseva.^23^ indicated that physical complaints, fear, anxiety and depression are higher in people experiencing unexpected events. Of course, there are also studies that, inconsistent with our findings, pointed out that the obsessive-compulsive disorder, depressive, anxiety, aggression, paranoid ideation, psychotic and fear disorders after an unexpected event in people are reduced with intervention and in the long-term and such affected people can return to a normal life and this important issue is determined only by the behavior pattern of humans.^24-27^ The researcher’s use of various tools for measuring various dimensions of psychological disorders when dealing with unforeseen events and the complexity of human behavior, and in some cases various sample sizes in different studies, may be the reasons for the diversity in the findings of studies in this field.

 Overall, our findings (Table 4) also showed that the average score of the overall psychological disorder in ISIS was statistically significantly different in comparison to the native population. In line with the present findings, Galovski andLyons,^28^ Barenbaum et al,^29^ Fegert et al,^30^ and Figley^31^ reported that suffering from psychological disorders as one of the frequent consequences of war trauma should be considered and effective and supportive intervention measures should be taken into account.

 The results (Table 5) demonstrated that although ISIS captives’ average GSI scores were higher than those of native people and there was a statistically significant difference between them, the average score of psychopathological symptoms in native people was still high. Perhaps one of the factors contributing to the observed high level of this index in native people is the crucial fact that, even though the native population was not captured, psychological disorders are still caused by war, the anxiety brought on by ISIS attacks, and the stress of being captured in comparison to the captive population, and this condition was indirect and unavoidable.

 The limitation of the present study was that the studied patients could not have a clinical evaluation; hence, the findings of the current study simply point to the existence of a psychiatric condition rather than providing a conclusive diagnosis. Another important limitation of this study was that statistical significance was considered more than clinical significance in the examination of differences. It means that sometimes the differences are small but significant, which may be due to the large sample size. We ask the readers of the article to consider the clinical significance of the differences in addition to the statistical significance.

## Conclusion

 In conclusion, the study’s findings indicate that ISIS prisoners experience a variety of psychological diseases, and because this demographic is more likely to experience more severe mental disorders, comprehensive psychiatric and psychological services are necessary for them.
